# Prevalence and trends of sexually transmitted infections among pregnant women in Mizan Tepi University Teaching Hospital, Southwest Ethiopia: a cross-sectional study

**DOI:** 10.11604/pamj.2022.42.111.30871

**Published:** 2022-06-09

**Authors:** Abiyot Wolie Asres, Mesenbet Muluken Endalew, Serawit Yirdaw Mengistu

**Affiliations:** 1 Department of Epidemiology and Biostatistics, School of Public Health, Wolaita Sodo University, Wolaita Sodo, Ethiopia,; 2 Mizan-Aman College of Health Science, Mizan-Aman, Ethiopia,; 3 Mizan Tepi University Teaching Hospitals, Mizan-Aman, Ethiopia

**Keywords:** Sexually transmitted infections, pregnancy, antenatal care, Ethiopia

## Abstract

**Introduction:**

sexually transmitted infections are the most common causes of illness in Africa. They are public health important diseases because of their magnitude, potential complications, and interactions with HIV/AIDS during pregnancy. In our country, especially in our study area, limited studies have been conducted to assess the magnitude and trends of sexually transmitted infections among pregnant women. Therefore, this study aimed to assess the prevalence and trends of sexually transmitted infections (STIs) among pregnant women.

**Methods:**

an institution-based cross-sectional study design was conducted in Mizan Tepi University Teaching Hospital in Southwest Ethiopia from August 1-30, 2019. Three hundred women were selected using a simple random sampling method from the women's registry book who visited the hospital for antenatal care (ANC) follow-up in the last five years. The data was collected by using checklists. Finally, the data were entered into Epi Info 7 and analyzed with statistical package for the social sciences (SPSS) version 25.0.

**Results:**

the prevalence of sexually transmitted infections was 50 (16.7%). HIV infection was 15 (5%), genital candidiasis 11 (3.7%), T. vaginitis 10 (3.3%), Hepatitis B virus 9 (3.0%), Hepatitis C virus 2 (0.7%), and Chlamydia 3 (1.0%). The trend of sexually transmitted infections over the last five consecutive years was increasing, decreasing, and again increasing.

**Conclusion:**

this study showed that the prevalence of sexually transmitted infections in pregnant women was relatively higher than in other similar studies conducted in different study areas. The trend of sexually transmitted infections in the last five years was not constant.

## Introduction

Sexually transmitted infections (STIs) are illnesses that have a significant probability of transmission from an infected person to healthy persons through unprotected sexual behavior, including vaginal intercourse, oral sex, and anal sex [1]. Sexually transmitted infections (STIs), including human immunodeficiency virus and acquired immune deficiency syndrome (HIV/AIDS), are an emerging public health concern, especially in developing countries. Globally, an estimated 1.3 billion people are suffering from STIs. Sexually transmitted infection rates tend to be higher for females than for males [2-4]. They predominantly affect young adults, facilitate transmission and acquisition of the human immunodeficiency virus (HIV) infection, and constitute a great socio-economic burden. Complications resulting from failure to diagnose and treat infections include pelvic inflammatory disease (PID), infertility, ectopic pregnancy, chronic pelvic pain, cervical cancer, and urethral stricture. The impact on fetuses and newborns can be devastating, as manifested by miscarriages, stillbirths, neonatal deaths, mental retardation, neonatal conjunctivitis, and pneumonia [5]. Having problems in the fetal period not only affects the health of the newborn but also has a major impact on adulthood [6]. Sexually transmitted infections are the most important public health diseases because of their magnitude, potential complications, and interaction with HIV/AIDS. The World Health Organization estimates that each year, more than 340 million new curable STIs occur in reproductive-aged men and women. This excludes the estimated 33 million new cases of HIV and the 100 million-plus infections caused by other viral STIs each year [7]. It was estimated that 500 million people were infected with syphilis, gonorrhea, chlamydia, or trichomoniasis. At least an additional 530 million people have genital herpes, and even 290 million women have human papillomavirus. STIs other than HIV resulted in 142,000 deaths in 2013 and were higher than this in the sub-Saharan Africa region [8]. Every year in Africa, approximately one million babies are stillbirth, and one million babies die in their first month of life. Infectious diseases and neonatal complications are responsible for the vast majority of these deaths. Sub-Saharan Africa remains the most affected region, with 25.6 million people living with HIV and approximately 8% carrying hepatitis B. In the absence of interventions, 30-45% of infants born to HIV-positive mothers in developing countries become infected during pregnancy, delivery, or breastfeeding, and it is estimated that only 54% of people living with HIV know their status [5,7,9,10].

Ethiopia is a country where STIs are highly prevalent [11]. In Ethiopia, as in other African countries, STIs are managed through syndrome case management, but data from public health facilities in Southern Ethiopia indicates that the quality of care for STIs was poor. There were no STI Syndromic management guidelines in all health facilities, and only 12.8% of clinicians had taken training on syndrome management [12]. In Ethiopia, HIV and syphilis were prevalent among pregnant women, indicating that they are significant public health problems. This indicates the need to enhance antenatal screening to reduce and ultimately prevent vertical and horizontal transmission of HIV and syphilis [1]. In Ethiopia, pregnant women are tested only once for HIV and syphilis during ANC visits using a rapid plasma regain (RPR) test, and those women reactive to RPR are treated immediately with one or more doses of intramuscular benzathine penicillin G 2.4 million units [13]. Despite the implementation of national STI prevention, care, and treatment strategies, programs, and guidelines in Ethiopia, the evidence from reviews of the studies conducted in different geographical locations in Ethiopia has shown that (HIV, Hepatitis B virus (HBV), syphilis, and herpes simplex 2 (HSV-2)) remain public health challenges in the country [8]. The high prevalence of STIs in pregnant women and their impact on the unborn child demonstrates the need for screening and treatment programs [14]. Sexually transmitted infections during pregnancy can have serious consequences for the mother and the infant. The study area is well-known for its mining resources, especially gold, so many people migrate from different parts of the country into this area. Most people, on the other hand, are pastoralists or nomadic. Hence, the knowledge, attitude, and practice of this population using STI preventive methods like condoms is very low. As a result, they are highly prone to having unsafe sexual intercourse and getting STIs, including HIV/AIDS. More importantly, the prevalence and trends of STIs in the area have not been well investigated. Therefore, this study aimed to assess the prevalence and trends of STIs among pregnant women who got ANC services at Mizan Tepi University Teaching Hospital.

## Methods

**Study design:** the institution-based cross-sectional study was carried out at Mizan Tepi University Teaching Hospital (MTUTH) in Bench Maji zone, Southwest Ethiopia, from August 1-30, 2019.

**Settings and population:** Mizan Tepi University Teaching Hospital is located in the Western Nations Nationalities region. It provides many services for the catchment areas of Bench Maj zone. The services have been provided in the gynecological and obstetrics, outpatient departments, in the medical department, surgical department, adolescent and youth-friendly units, TB/HIV/AIDS clinic, and in the pediatrics department and other emergency and special care service units. The source populations were all pregnant women who came to MTUTH in the last five years. All pregnant women who received ANC services at MTUTH from June 2014 to June 2019 were included in the study. The individual ANC follow-up card of a pregnant woman was the study unit.

**Sample size determination:** the required sample size was determined by using a single population proportion formula considering the following assumptions: proportion = 26.6% [11], level of significance= 95%, and the margin of error = 5%. Hence, the final sample size was 300.

**Sampling procedures:** we used an adapted and validated checklist to collect the data. The instruments (checklists) were adapted from the study conducted in Gondar town [14]. A sampling frame (using five-year ANC registration books) was used. All cards of pregnant women who visited the ANC clinic within the period of June 2014 to June 2019 were included in the study. After allocating samples proportionally for the last five years, 300 study subjects (ANC cards) were selected by using a simple random sampling method from each year.

**Data quality assurance:** data quality has been ensured by using an adapted valid checklist during collection. In addition, trained data collectors, supervisors, and principal investigator have checked the data consistency and completeness every day.

**Data analysis:** the data were cleaned, edited, checked, and entered into EPI info 7 and analyzed by using SPSS version 25.0. The findings were presented with frequency distributions and percentages by using texts, tables, and figures.

**Ethical consideration:** ethics approval was obtained from the Institution Review Board of Mizan Tepi University. Before, data collection, permission was received from the card and data room offices. The confidentiality of all information from the individual cards was kept.

## Results

**Sociodemographic characteristics:** a total sample size of 300 medical records (ANC cards) of women who had ANC follow-up were included. Out of these, 120 (40%) pregnant women were in the age range of 25-29 years, and the mean age was 24.91±4.35 SD years. Almost all 296 (98.7%) women were married, and two-thirds of 190 (63.3%) lived in urban areas (Table 1).

**Table 1:** frequency distribution of sociodemographic characteristics of women attended ANC

Variable	Categories	Frequency	Percentage
**Age**	15-19	32	10.7
	20-24	98	32.7
	25-29	120	40.0
	30-34	39	13.0
	35-39	11	3.7
	Total	300	100.0
**Marital status**	Single	2	0.7
	Married	296	98.7
	Divorced	2	0.7
	Total	300	100.0
**Place of residence**	Urban	190	63.3
	rural	110	36.7
	Total	300	100.0

**General descriptive characteristics**: about 70 (23.3%) pregnant women were primigravida, around 124 (41.3%) were in their second pregnancy, and one-third, 106 (35.3%), were multigravida, and 49 (16.3%) had at least one history of abortion. Of the total pregnant women in the study, more than half (58.7%) were reached at their fourth ANC visit, and 23 (7.7%) were at their first visit (Table 2).

**Table 2:** frequency distribution of women who attended ANC services in Southwest Ethiopia

Variables	Categories	Frequency	Percentage
Number of gravida	Gravida one	70	23.3
	Gravida two	124	41.3
	Gravida three	53	17.7
	Gravida four	29	9.7
	Gravida five	11	3.7
	Gravida six	13	4.3
	Total	300	100.0
Number of parity (n=211)	Para 1	114	54.0
	Para 2	55	26.0
	Para 3	18	8.5
	Para 4	15	7.1
	Para 5 and above	9	4.3
	Total	211	100.0
History of abortion	Yes	49	16.3
	No	251	83.7
	Total	300	100.0
Type of abortion for yes only (n=49)	Spontaneous	49	100.0
	Total	49	100.0
Number of ANC visits	1^st^ visit	23	7.7
	2^nd^ visit	25	8.3
	3^rd^ visit	76	25.3
	4^th^ visit	176	58.7
	Total	300	100.0

**Figure 1:**
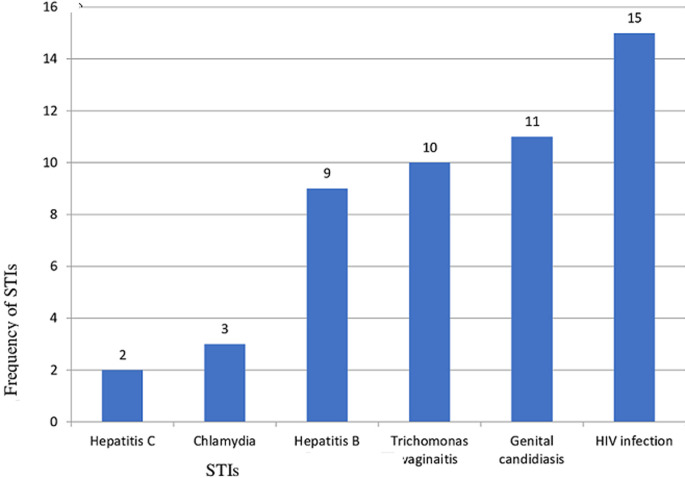
types of sexually transmitted infections (STIs) among pregnant women who received antenatal care service in Southwest Ethiopia

**Figure 2:**
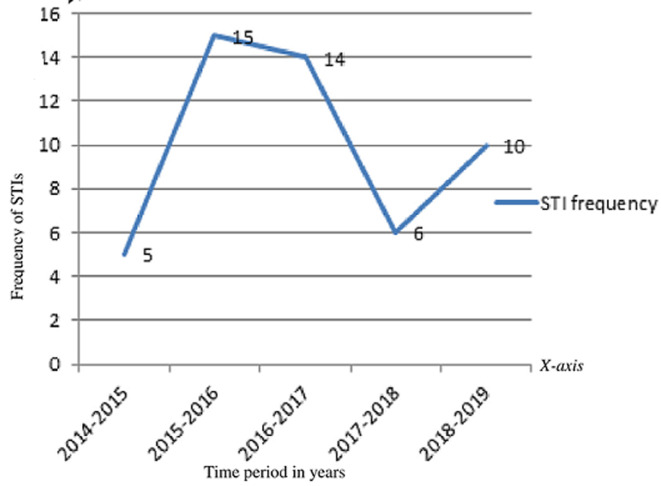
trend of sexually transmitted infections (STIs) over the last five years among pregnant women who attended antenatal care

**Prevalence and trends of STIs:** according to this study, the prevalence of STIs was 50 (16.7%). From this, HIV infection was 15 (5.0%), genital candidiasis 11 (3.7%), *T. vaginitis* 10 (3.3%), hepatitis B virus 9 (3.0%), Chlamydia 3 (1.0%) and hepatitis C virus 2 (0.7%) (Figure 1). The findings from the medical record review of the cards of ANC-followed mothers revealed that, overall, 50 (16.7%) study subjects were diagnosed with STIs. In this study, the trend of the prevalence of sexually transmitted infections over the last five years was not constant. The year with the highest prevalence was 2015, and the year after that was also 2016. Since then, the prevalence has decreased. The least prevalence recorded was in 2014. Generally, it has varied in an increasing to decreasing manner. It had primarily increased during the first three years, then declined in the middle of the fourth year, and then increased again in the fifth year (Figure 2).

## Discussion

In the current study, the prevalence of STIs was 50 (16.7%). The prevalence of STIs in the current study was high as compared with studies done in Latin America (4.6%), Asia (11.2%), and the four African countries (7.7%) [15-17]. The variation may be due to the fact that, in the current study, most people were living as pastoralists, had low knowledge of STI prevention methods, and even had no access to getting these methods. Due to the presence of gold, many people migrated to this area, and it has a dense population. Hence, they are more prone to risky sexual practices and acquiring STIs, including HIV/AIDS. In addition to this, the variation may be due to the accuracy of test screening, method differences, or sample size differences. In other study areas, however, they have better access to STIs, including HIV/AIDS prevention methods, and have better knowledge and practice with these methods. Furthermore, the study designs in these previous studies were systematic review and meta-analysis, which may give more pooled prevalence results. The prevalence of the current study was also higher than the study conducted in Nepal, where the overall prevalence of any STIs was 8.6%, 1.5% for *Chlamydia trachomati* (CT), and 7.1% for trichomoniasis infection [18]. In Nepal, the study was in a semi-urban population where more resources are available, and the people may get better information access regarding STIs as compared with the current study area. However, the prevalence of the current study was comparable with a study conducted in the Gambia where the overall prevalence of STIs was 53.6%. Specifically, HIV was (5.7%), *T. vaginitis* (3.9%), *Neisseria gonorrhoeae* (*N. gonorrhea*) (1.8%) and *Chlamydia trachomatis* (*C. trachomatis*) (0.7%) [19]. In contrast, the prevalence of STIs in this study was lower as compared to a study conducted in Northern Tanzania (54.3%), of which 7% were HIV infections, 0.9% active syphilis, 23.9% bacterial vaginosis, 17.5% *Chlamydia trachomati*, and 0.5% *Neisseria gonorrhoeae* [9,10].

Differences in laboratory materials' specificity and sensitivity tests, lab technicians' skill, limited sample size, sociodemographic diversity, differences in the study population, population lifestyle, or community health-seeking behavior could all contribute to the inconsistencies. The study populations in a study conducted in Tanzania were pregnant women in third trimester and above, but in the current all pregnant women at any trimester were included. This may affect the result which means after the third trimester most STIs show symptoms but in the early pregnancy they might be asymptomatic. On the other hand, the prevalence of STIs in this study was higher than compared to the studies conducted in Adami Tulu woreda and Ayder Referral hospital of Mekele town, which were (3.3%) and (8.5%), respectively [8, 10]. The discrepancy might be due to the study design and population differences; that is, the study conducted in Adami Tulu woreda was community-based, while the study carried out in Ayder referral hospital, Mekelle, was focused on only pregnant women on antiretroviral therapy (ART) follow-up. Similarly, in the current study area, there is a possibility of being married or having more than one housewife in a culture in which having more than one sexual partner is the main cause of developing STIs. The accessibility, affordability, knowledge, attitude, and practice of using the STI prevention methods are very different when compared with the current study area and that of these two towns. The lifestyle is also different in these areas. The trend of STIs in the current study was intermittent. The finding was in contrast with a study done in Ethiopia, where the trend of STIs declined by > 44% [20]. This may be due to the sample size and the fact that the study area for the current study is limited as compared to that of the previous. Similarly, it might be due to a difference in the study period. The limitation of the current study is that it may not show the exact trends of STIs in the study area due to the small sample size each year and the cross-sectional nature of the study.

## Conclusion

The prevalence of sexually transmitted infections in the current study was relatively higher than in other similar studies conducted in other study areas. The trend of STIs over the last five years has been intermittent in the MTUTH. This indicates the need to enhance antenatal STI screening services to reduce and ultimately prevent the health of mothers and fetuses. Appropriate strategies should be advised for the prevention and control of sexually transmitted diseases in pregnant women, above all in females of reproductive age. Therefore, it is an alarming issue and the concerned bodies should give or take corrective measures because STIs are major public health problems.

### What is known about this topic

- Pregnant women are easily infected by sexually transmitted infections;
- Sexually transmitted infections facilitate the progression of HIV/AIDS;
- Most sexually transmitted infections are treatable with antibiotics.

### What this study adds

- The current prevalence of sexually transmitted infections in the study area is very high;
- Sexually transmitted infections trends over the past three years have been inconsistent;
- It will serve as input for other researchers.
